# Assessment of Psychometric Characteristics of Parkinson’s Disease Sleep Scale 2 and Analysis of a Cut-Off Score for Detecting Insomnia in Italian Patients with Parkinson’s Disease: A Validation Study

**DOI:** 10.3390/jpm14030298

**Published:** 2024-03-10

**Authors:** Claudio Liguori, Francesco Frontani, Giulia Francescangeli, Mariangela Pierantozzi, Rocco Cerroni, Tommaso Schirinzi, Alessandro Stefani, Nicola Biagio Mercuri, Giovanni Galeoto

**Affiliations:** 1 UOSD Parkinson, Neurology Unit, University Hospital of Rome Tor Vergata, 00133 Rome, Italy; claudioliguori@yahoo.it (C.L.); pierantozzim@gmail.com (M.P.); rocco.cerroni@gmail.com (R.C.); t.schirinzi@yahoo.com (T.S.); stefani@uniroma2.it (A.S.); mercurin@med.uniroma2.it (N.B.M.); 2 Department of Systems Medicine, University of Rome Tor Vergata, 00133 Rome, Italy; francescangeligiulia@gmail.com; 3 Departmental Faculty of Medicine and Surgery, Saint Camillus International University of Rome and Medical Sciences (UniCamillus), 00131 Rome, Italy; 4 Department of Human Neurosciences, Sapienza University of Rome, 00185 Rome, Italy; giovanni.galeoto@uniroma1.it; 5 Neuromed IRCCS, 86077 Pozzilli, Italy

**Keywords:** Parkinson’s disease, sleep disorder, non-motor symptom, questionnaire

## Abstract

Introduction: Sleep disorders are frequent non-motor symptoms affecting patients with Parkinson’s disease (PD). Insomnia represents the most common sleep disorder. Parkinson’s disease Sleep Scale 2 (PDSS-2) is a specific tool to investigate sleep problems in PD. The General Sleep Disturbances Scale (GSDS) was a general scale validated for the Italian population. Our goal was to assess the psychometric characteristics of PDSS-2 and the GSDS in this population, calculating a cut-off score for insomnia symptoms by using subitems of PDSS-2. Methods: Patients admitted at the PD Unit of the Hospital of Rome Tor Vergata outpatient clinic and those afferent to PD associations were asked to complete PDSS-2 and GSDS to be correlated to identify a cut-off for insomnia symptoms. Items 1,2,3,8,13 of PDSS-2 were used to detect insomnia. An ROC curve to assess a cut-off score for insomnia was determined. A cross-cultural analysis of PD population characteristics was performed. Results: In total, 350 PD patients were recruited. Cronbach’s alpha was high for the total score (0.828 for PDSS-2 and 0.832 for GSDS). A cross-cultural analysis did not show any significant *p*-value. The ROC curve yielded an AUC of 0.79 (CI: 0.75–0.84). The cut-off value for insomnia disorder based on items 1,2,3,8,13 of PDSS-2 was >10, demonstrating a sensitivity of 76% and a specificity of 69% in determining the presence of subjective insomnia symptoms in PD. Discussion: PDSS-2 is demonstrated to be a valid, specific tool to address sleep disturbances in PD patients. A cut-off score of 10 for items 1,2,3,8,13 was identified for detecting insomnia symptoms in PD patients.

## 1. Introduction

Parkinson’s disease (PD) is characterized by bradykinesia, rigidity, and tremors as main motor symptoms, but in the recent past, non-motor symptoms have emerged as important determinants of patients’ well-being. PD represents a frequent cause of morbidity that affects 1–2 per 1000 of the population at any time, clearly most often in the older age groups [1]. The prevalence of PD is about 1% in people over 60 years of age [2]. Considering the non-motor symptoms, sleep disturbances are estimated to occur in 60–98% of PD patients, affecting all stages of the disease and increasing the prevalence in motor fluctuating patients; moreover, non-motor symptoms may have important implications in the management and prognosis of PD [3,4]. Recently, Fernandes et al. suggested the increasing need to consider the non-motor symptoms in clinical practice and their different prevalence across the stages of the disease, the influence of gender, and the clinical PD characteristics [4]. Insomnia, excessive daytime sleepiness (EDS), sleep-disordered breathing (SDB), circadian sleep–wake cycle disorders, restless legs syndrome (RLS), and REM sleep behavior disorder (RBD) are the main sleep disorders affecting PD patients [5]. Sleep disorders negatively impact PD patients’ well-being [6]. Insomnia is closely related to depression and lower health-related quality of life [7,8]; moreover, insomnia is frequently associated with other sleep disorders such as Obstructive Sleep Apnea (OSA), RBD, and RLS [9]. EDS is associated with increased motor disability, cognitive impairment, and worse quality of life [9,10]. SDB, as well as insomnia and EDS, is associated with cognitive dysfunction [11]. Circadian sleep–wake rhythm disorders significantly affect patients’ activities of daily living, motor impairment, and treatment schedules [12]. RLS is commonly associated with periodic limb movements in sleep (PLMS), and consequently, it can also affect sleep latency and continuity [9]. Increasing evidence indicates that RBD may be commonly associated with some other non-motor symptoms including olfactory dysfunction; color vision impairment, such abnormal color vision discrimination; depression; cognitive abnormalities; and EDS [13]. Therefore, considering the importance of sleep disturbances in managing PD patients, screening for them to set therapeutic strategies becomes a standard of care for clinicians. The Parkinson’s disease Sleep Scale version 2 (PDSS-2) is a scale specifically designed to assess sleep problems in PD patients [14] and has been validated in several languages: Hindi [15], Persian [16], Korean [17], Spanish [18], and Italian [19]. The scale shows an excellent psychometric property across languages, with Cronbach’s alpha values of 0.73 (English) [20], 0.8 (Hindi), 0.9 (Persian), 0.85 (Korean) , 0.84 (Spanish), and 0.77 (Italian). PDSS-2 showed a sensitivity of 77.6% and a specificity of 74.3% in the clinical assessment of PD-specific sleep problems. According to a physician assessment, Parkinson’s disease-specific sleep disturbances occur in 62% of patients [21]. No correlation has been assessed between PDSS-2 and other scales. Therefore, the first aim of this study is to assess the psychometric characteristics of PDSS-2, find out if there are differences among the population considering different bands of age and sex, and finally determine a cut-off score for detecting insomnia in PD patients using the PDSS-2 cluster of items which are considered to be related to insomnia [20,22] (as suggested by Trenkwalder et al. 2011 and Suzuki at al 2015)) in order to find out a score of the PDSS-2 that could be helpful for clinicians in assessing different sleep disorders. Considering that the treatment of sleep disorders needs to be tailored individually according to the predominant clinical symptomatology and underlying specific sleep-related diagnosis [5], secondly, we assess this population’s psychometric characteristics of the General Sleep Disturbances Scale (GSDS). The GSDS is a useful tool for assessing the presence of sleep disorders in the general population and has been validated in Italian [23] in various pathologies such as spinal cord injuries [24], hip replacement [25], and autistic spectrum disorder [26].

## 2. Materials and Methods

### 2.1. Participant

The participants were recruited thanks to the collaboration of different territorial associations that follow patients with PD, as well as the patients of the PD center of the University of Rome Tor Vergata between September and November 2022. The inclusion criteria were to be aged more than 18 years old and have an idiopathic PD diagnosis. The diagnosis was performed following the criterion made by the UK Parkinson’s disease Society Brain Bank [27]: comprehending and compiling the questionnaire and giving consent to the study. We administered the questionnaire in two different ways, with the online form and the same form administered by one researcher. The online questionnaire was performed first, and the subjects involved were asked to compile the questionnaire physically again in the next 72 hours. An ethics committee was unnecessary for this type of study. All procedures followed were in accordance with the ethical standards of the committee responsible for human experimentation (institutional and national) and with the Helsinki Declaration of 1975, as revised in 2008. The number of the ethical protocol was Prot. 0428/2020, obtained in 10 June 2020 by the University ‘’SAPIENZA’’ commission.

### 2.2. Outcome Measure

Different questionnaires were administered to the population. The online form comprised some questions to investigate the patient characteristics (age, sex, diagnosis), the PDSS-2, and the GSDS. The patients were divided into two categories, considering the Diagnostic and Statistical Manual of Mental Disorders fifth edition (DSM-5), as the ones who suffered from insomnia and the ones who did not suffer from insomnia to assess the discriminant validity to determine a cut-off score for the GSDS in this population for this specific sleep disorder [23,28]. To reach a diagnosis of insomnia disorder, the following criteria needed to be met: unhappiness with the quality or quantity of sleep, which can include trouble falling asleep, staying asleep, or waking up early and being unable to fall back asleep; the sleep disturbance causes significant distress or impairment in functioning, such as within the individual’s working or personal life, behaviorally, or emotionally; difficulty sleeping, occurring at least three times a week and present for at least three months; the problem occurs despite ample opportunity to sleep; the difficulty cannot be better explained by other physical, mental, or sleep–wake disorders; and the problem cannot be attributed to substance use or medication. This methodology was previously used in another study [23].

### 2.3. PDSS-2

The PDSS-2 is a self-administered questionnaire composed of 15 items. The score ranged the frequency of sleep disturbance in the last week from 0 (never) to 4 (very often). It was translated and tested in Italian, showing a high Cronbach’s alpha (0.77) and high test–retest reliability (0.943) [19]. Moreover, the authors identified 5 different factors related to different components of sleep disturbance and compiled them based on the combination of the items of the scale: factor 1, motor symptoms; factor 2, sleep quality; factor 3, dreaming distress; factor 4, fragmented sleep; factor 5, insomnia symptoms [19].

### 2.4. GSDS

The GSDS is a self-administered scale composed of 21 items divided into 6 domains: 1, difficulty in falling sleep; 2, waking up during sleep; 3, quality of sleep; 4, quantity of sleep; 5, daytime sleepiness; 6, use of substances to help induce sleep. The score ranges from 0 (never) to 7 (every day) and refers to the last week’s sleep disturbances. It was translated and tested in Italian, showing a high Cronbach’s alpha (0.77) and high test–retest reliability (0.78) [23]. Moreover, the authors investigated the cut-off score of 38.5, with a sensitivity of 75% and a specificity of 80%.

### 2.5. Reliability

Internal consistency is defined as the extent to which items in a subscale are homogeneous, thus measuring the same concept [29]. The internal consistency of the PDSS-2 was analyzed using the Cronbach alpha, considering the total score of the questionnaire. In the same way, the GSDS internal consistency was analyzed. A Cronbach alpha between 0.7 and 0.95 was considered satisfactory [29]. It was assessed using every questionnaire that was filled out completely by the subjects included in this study.

### 2.6. Construct Validity

Concurrent validity was analyzed using Pearson’s correlation to determine the PDSS-2 and GSDS association. The correlation was considered strong at over 0.7, moderate between 0.3 and 0.7, and scarce when less than 0.3 [30]. This procedure was performed to investigate the capability of a general questionnaire to analyze a specific population. It was assessed using every questionnaire that was filled out completely by the subjects included in this study.

### 2.7. Cut-Off Score of PDSS-2

The Receiver Operating Characteristic (ROC) curve and the area under the curves (AUCs) were created and analyzed to compare PDSS-2 items 1,2,3,8,13, as suggested by Trenkwalder et al. 2011 and Suzuki at al 2015 [20,22], for detecting insomnia symptoms and data on sleep disturbances. The cut-off point, sensitivity, and specificity were identified using Youden’s index [31]. The AUC refers to precise data, while imprecise data shows a 0.50 AUC. Usually, an AUC higher than 0.75 shows moderate scale predictors, while excellent ones are obtained with an AUC ≥ 0.90 [32]. It was assessed using every questionnaire that was filled out completely by the subjects included in this study.

### 2.8. Score Analysis

We performed an ANOVA test for more samples to detect a demographic characteristic of the sample that could modify the questionnaire score. The *p*-value was set at 0.05. The total score of the PDSS-2 was matched with sex- and age-band groups.

### 2.9. Statistical Analysis

All statistical analyses were performed using IBM-SPSS version 23.00.

## 3. Result

### 3.1. Participants

In total, 441 PD patients were included in this study, and the analysis was performed on a group of 350 patients, since 91 were excluded because of incomplete data; 48.3% of female patients constituted the sample. Just 10% of our sample were under 50 years old and showing early-onset PD, while just 6.6% of the patients were older than 80 years old. Overall, 61.4% of the patients were not affected by insomnia disorder. In Table 1, the main characteristics of the group are represented.

### 3.2. Reliability

The reliability of the PDSS-2 was found to be high, with an alpha deleted score that ranged from 0.806 to 0.845. The total score’s alpha was 0.828. The lower Cronbach’s alpha of PDSS-2 was shown by the fifth item (0.806), while the higher score was from the fourth item (0.845) (Table 2). The reliability of the GSDS was found to be high, with a score that ranged from 0.806 to 0.867. Also, the total score presents a high reliability, with a Cronbach’s alpha = 0.832 (Table 3). The lower Cronbach’s alpha of the GSDS was shown by the nineth and eleventh items (0.806), while the higher score was from the fourth item (0.867).

### 3.3. Construct Validity

The construct validity was obtained by comparing the PDSS-2 and GSDS. The comparison between the total score of the two questionnaires was moderate (r = 0.615). A scarce correlation was found between subscale 3 of the GSDS and the PDSS-2, with a score that ranged from −0.047 to 0.164, as well as a significant *p* only for PDSS-2 subscales 4 and 5; subscale 6 of PDSS-2 had a significant *p* for all analyses, but a score which ranged from 0.151 to 0.29. Two comparisons showed an inverse correlation between two scales: the comparison between subscale 3 of the GSDS and subscales 2 and 3 of the PDSS-2. A moderate correlation was analyzed between the subscales and the total score of the two questionnaires, with an r that ranged from 0.3 to 0.637 (Table 4).

### 3.4. Score Analysis

**Figure 1:**
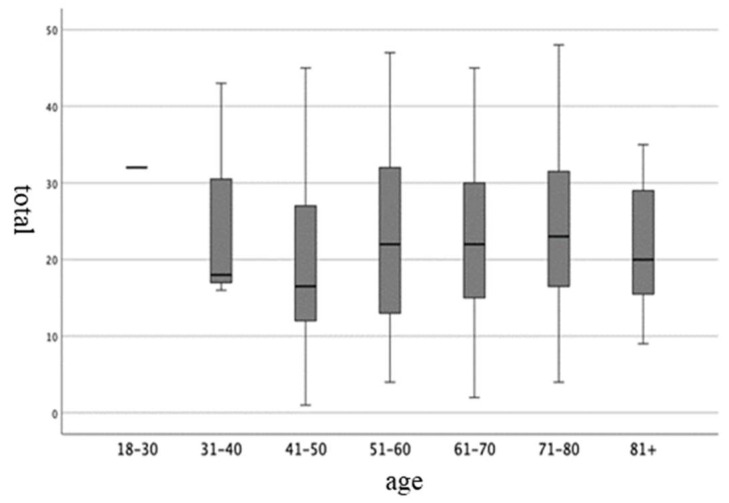
Graphical representation of the results shown in Table 5.

**Figure 2:**
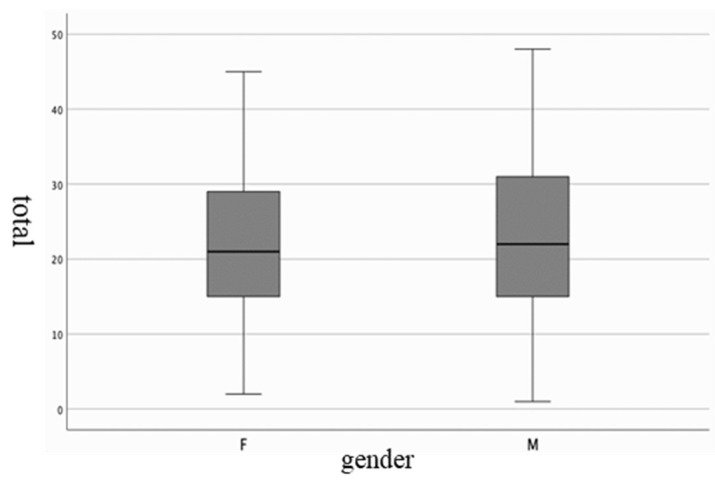
Graphical representation of the results shown in Table 6.

The cross-cultural analysis compared the PDSS-2 with the sample age and sex. No significant differences were found between groups. The results of the mean, standard deviation (SD), median, and variance are shown in Table 5 and Figure 1. The lowest score was shown by the age band between 41 and 50 (18.27). Sex analysis did not show any differences between groups; the variance shown by the female sex was 109,821, with 114,283 for the male sex. Table 6 and Figure 2 report the results of the analysis.

### 3.5. Cut-Off Score

**Figure 3:**
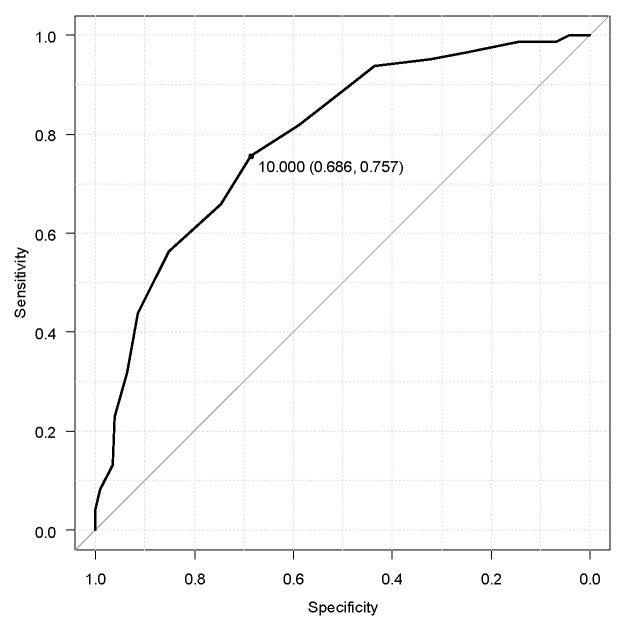
ROC curve to analyze the cut-off score of the PDSS-2 items 1,2,3,8,13 for detecting insomnia.

The ROC curve analysis showed that predicting the presence of sleep disturbances yielded an AUC of 0.79 (CI: 0.75–0.84). The cut-off value of the combination of items 1,2,3,8,13 of the PDSS-2 was >10, demonstrating a sensitivity of 76% and a specificity of 69% for detecting insomnia symptoms. The value was obtained from a stratification through the DSM-5. The results are graphically shown in Figure 3.

## 4. Discussion

The first aim of this study was to confirm and enrich the evaluation of the statistical properties of the PDSS-2, including a cut-off score for insomnia disturbances in subjects with PD. PDSS-2 has been extensively used in PD and is thus recommendable for screening and measuring severity of sleep disturbances in PD; however, clinical cut-off values regarding the different sleep disorders are still missing [33]. We administered the scale to a huge sample of 441 patients; however, due to missing data, we could only obtain data from 350 PD patients. The reliability of the total score was alpha = 0.828. This value was higher than the one shown in the original study [22] and the other Italian validation; maybe the high magnitude of the sample affected it. However, these other studies also showed high Cronbach’s alpha reliability: 0.77 for the previous Italian validation and 0.73 for the original study. Moreover, our data were in line with the validation of the PDSS-2 in other languages: Hindi (0.8), Persian (0.9), Spanish (0.84), and Korean (0.85). Considering the score analysis, in which we compared the scores reached for different sex and age bands, we did not find a statistically significant difference considering sex or age in our sample. The PD patients aged between 31 and 40 reached a higher mean score (25.67) in the questionnaire, followed by patients aged 71–80 (24.11). Other studies found that the earliest-onset and late-onset patients with PD can present with sleep disturbances, as well as the oldest patients with PD [34,35], and our results were consistent with the previous literature. This element seems relevant considering that PD patients reporting poor sleep are those at high risk of developing depression [36,37], a decrease in quality of life [6], and cognitive impairment [38], among others. So, addressing more carefully the sleep disorders in these two age bands is required. Moreover, as recently shown, nocturnal disturbances likely increase in PD patients, considering the disease severity and duration and not only considering the patient’s age. No differences were found considering sex, with similar scores in men and women; the mean value for men was 22.82 (SD: 10.69), with 22.54 (SD: 10.48) for women, showing no differences at all between the two genders, and these data are consistent with those previously shown in a cross-sectional study on a Chinese population [39]. For clinicians and researchers, determining a cut-off score for screening symptoms using a questionnaire is important. Considering our sample, 135 subjects were affected by insomnia disorder (38.4% of the sample). We analyzed the ROC curve and the AUC using items 1-2-3-8-13 of the PDSS-2, which have been related to insomnia symptoms. The result showed a cut-off score of >10, with a sensitivity of 76% and a specificity of 69%. These data could be interesting for clinicians during the assessment of different disorders related to PD. Finding a cut-off score for the questionnaire based on its subscale could help the clinicians in screening and thus provide the proper treatment without using or proposing more expensive assessment tools or allowing clinicians to choose patients who need further investigation by analyzing the results from the PDSS-2. This value was interesting compared to that previously shown in the literature [20]. Muntean et al. found a cut-off value of 18 for the total scale considering all the clinically relevant sleep disorders. Suzuki et al. showed a cut-off score of 15 considering the total score of the PDSS-2, which could be helpful to define good and poor sleepers. The values shown in our analysis were lower than others shown previously in the literature. However, we focused on a specific cluster of five items to assess a specific sleep disorder such as insomnia. Insomnia was shown to be one of the most frequent sleep disorders in PD patient, with 83% of patients with PD reporting symptoms of insomnia [40]. So, the cut-off value retrieved in this study in assessing PD patients could help to differentiate between different sleep disorders in patients who do not show a total score higher than 15 [22] or 18 [21]. Differentiating between sleep disorders that affect PD patients is fundamental, considering that different disorders need tailored treatments. The treatment in the case of insomnia is generally based on hypnotics, quetiapine or clozapine in severe cases of insomnia [5]. These data could help clinicians to address the proper treatment for their patient. This study’s second aim was to assess the psychometric properties of the GSDS, a tool to assess sleeping disorders in the general population, to analyze their pertinence in this specific population. The GSDS showed a high score for Cronbach’s alpha in this population, ranging from 0.806 and 0.867 for the alpha item deleted and 0.832 for Cronbach’s alpha total score. So, the GSDS questionnaire showed a strong reliability in this population. Another fundamental aspect of using a psychometric scale is construct validity. To analyze the construct validity, we correlated the PDSS-2 and GSDS. A scarce correlation was found between subscales 3 and 6 of the GSDS, all subscales of the PDSS-2, and the total score, in which all scores were lower than 0.3. These two subscales of the GSDS analyzed sleep quality in general and the use of drugs to help induce sleep. The use of drugs is not analyzed in PDSS-2, so the low correlation between the two scales was expected and could be considered appropriate. Generally, the results demonstrated a moderate correlation between the subscales and total scores of the PDSS-2 and GSDS, with a score that ranges from 0.3 to 0.637. Pearson’s analysis of the two scales showed that none of the items analyzed reached a strong correlation between the two scales. However, the correlation between the total scores of the two questionnaires was moderate (r = 0.615) and it showed one of the highest results in the comparison between the two scales. We did not expect such a difference between the two scales, and we suppose that some subscales could reach better results, showing a strong comparison between the tools. So, considering these results, the GSDS, which is a general questionnaire that provides interesting data on different categories of patients, like hip replacement patients and spinal cord injury patients, did not provide good construct validity compared to PDSS-2, and considering our results, we do not suggest to use it on PD patients. These data highlight the importance of using, when it is possible, a specific questionnaire when assessing a population with specific disorders. Moreover, we again showed the importance of analyzing the different psychometric properties of a scale for every specific disorder, because the characteristic of a questionnaire and its capability to properly assess a population could change when modifying the sample of interest.

## 5. Conclusions

Both PDSS-2 and the GSDS showed high reliability in assessing insomnia symptoms in PD patients. The cut-off score to identify insomnia using items 1-2-3-8-13 of PDSS-2 was set at >10, with a sensitivity of 69% and a specificity of 76%. The score analysis showed that early-onset patients (31–40 years old) seem to suffer from sleep disturbances more than the other groups, but without showing a significant difference between them and other age-band groups. No sex-based differences emerged in the comparison between men and women. Finally, a moderate to scarce correlation between the PDSS-2 questionnaire and the GSDS questionnaire was shown, highlighting the importance of using a specific questionnaire when it is possible.

## Figures and Tables

**Table 1:** Sample characteristics.

	Sample *N* = 350	
Age	N°	%
18–30	1	0.3
31–40	3	0.9
41–50	30	8.6
51–60	73	20.9
61–70	121	34.6
71–80	99	28.3
81+	23	6.6
Sex	N°	%
F	169	48.3
M	181	51.7
Insomnia Disorder	N°	%
No	215	61.4
Yes	135	38.6

**Table 2:** Mean ± (SD), alpha (item deleted) and Cronbach’s alpha total score of the PDSS-2.

	Mean	Standard Deviation	Alpha Deleted	Cronbach’s Alpha
item 1	1.87	1.29	0.815	
item 2	1.47	1.37	0.821	
item 3	2.27	1.34	0.811	
item 4	1.42	1.39	0.845	
item 5	1.21	1.37	0.806	
item 6	1.36	1.29	0.815	
item 7	0.55	1	0.819	
item 8	2.61	1.29	0.829	
item 9	1.83	1.51	0.815	
item 10	1.32	1.36	0.806	
item 11	1.35	1.25	0.812	
item 12	1.58	1.37	0.810	
item 13	0.97	1.27	0.828	
item 14	2.19	1.22	0.814	
item 15	0.70	1.03	0.820	
Total score				0.828

**Table 3:** Mean ± (SD), alpha deleted score and Cronbach’s alpha total score of the GSDS.

	Mean	Standard Deviation	Alpha Deleted	Cronbach’s Alpha
Item 1	2.2	2.38	0.823	
Item 2	4.86	2.41	0.823	
Item 3	3.92	2.57	0.821	
Item 4	2.72	2.26	0.867	
Item 5	3.48	2.55	0.812	
Item 6	4.03	2.33	0.813	
Item 7	2.56	2.46	0.816	
Item 8	2.64	2.3	0.817	
Item 9	4.19	2.28	0.806	
Item 10	4.12	2.32	0.808	
Item 11	4.11	2.33	0.806	
Item 12	4.15	2.3	0.813	
Item 13	3.78	2.57	0.819	
Item 14	2.83	2.62	0.841	
Item 15	1.83	2.14	0.829	
Item 16	0.23	0.93	0.834	
Item 17	0.22	1.11	0.835	
Item 18	0.95	2.12	0.833	
Item 19	0.33	1.34	0.834	
Item 20	2.06	3.06	0.835	
Item 21	0.47	1.46	0.831	
Total score				0.832

**Table 4:** Correlation between PDSS-2 and GSDS. ** *p* < 0.001.

	PDSS-2					
GSDS	Subscale 1	Subscale 2	Subscale 3	Subscale 4	Subscale 5	Total
Subscale 1	0.292 **	0.447 **	0.214 **	0.394 **	0.595 **	0.500 **
Subscale 2	0.375 **	0.568 **	0.293 **	0.637 **	0.332 **	0.596 **
Subscale 3	0.005	−0.047	−0.041	0.157 **	0.163 **	0.058
Subscale 4	0.268 **	0.515 **	0.238 **	0.346 **	0.286 **	0.432 **
Subscale 5	0.315 **	0.538 **	0.387 **	0.413 **	0.300 **	0.516 **
Subscale 6	0.151 **	0.290 **	0.194 **	0.218 **	0.216 **	0.275 **
Total	0.372 **	0.600 **	0.402 **	0.548 **	0.402 **	0.615 **

**Table 5:** Mean ± (SD), median, and variance score of PDSS-2 for the different age band groups.

	Age Band					
	31–40	41–50	51–60	61–70	71–80	81+
Mean ± (SD)	25.67 (15.04)	18.27 (10.16)	22.93 (11.56)	22.59 (10.37)	24.11 (10.47)	21.22 (7.75)
Median	18.00	16.50	22.00	22.00	23.00	20.00
Variance	226,333	103,306	133,648	107,711	109,712	60,178

**Table 6:** Mean ± (SD), median, and variance score of PDSS-2 considering the sex.

	F	M
Mean ± (SD)	22.54 (10.48)	22.82 (10.69)
Median	21.00	22.00
Variance	109,821	114,283

## Data Availability

All data generated or analyzed during this study are included in the published article.
